# Tackling the mangrove restoration challenge

**DOI:** 10.1371/journal.pbio.3001836

**Published:** 2022-10-17

**Authors:** Catherine E. Lovelock, Edward Barbier, Carlos M. Duarte

**Affiliations:** 1 School of Biological Sciences, The University of Queensland, St Lucia, Queensland, Australia; 2 Department of Economics, Colorado State University, Fort Collins, Colorado, United States of America; 3 King Abdullah University of Science and Technology (KAUST), Red Sea Research Center (RSRC), Thuwal, Saudi Arabia; Smithsonian Institution, UNITED STATES

## Abstract

Mangroves have been converted and degraded for decades. Rates of loss have declined over the past decades, but achieving resilient coastlines requires both conservation and restoration. Here, we outline the challenges for the global restoration of mangroves and what actions could enhance restoration. Ambitious global targets for mangrove restoration, if successful, could deliver global benefits of carbon sequestration, fisheries production, biodiversity, and coastal protection. However, large-scale mangrove planting efforts have often failed, and smaller projects may not deliver landscape-scale benefits, even though they are more suited to community management. Solutions to achieving global targets include reducing risks of large projects and increasing the uptake and effectiveness of smaller projects. Sustainable mangrove restoration requires investment in capacity building in communities and institutions, and mechanisms to match restoration opportunities with prospective supporters and investors. Global reporting standards will support adaptive management and help fully understand and monitor the benefits of mangrove restoration.

## Abstract 2

Restoration of mangroves is urgently needed and contributes to climate change mitigation, but often faces biophysical, social, economic and regulatory barriers. This Essay describes emerging solutions supporting restoration of mangroves - solutions that are needed to fully implement restoration goals and achieve resilient, sustainable coastal communities.

## Introduction

**Fig 1:**
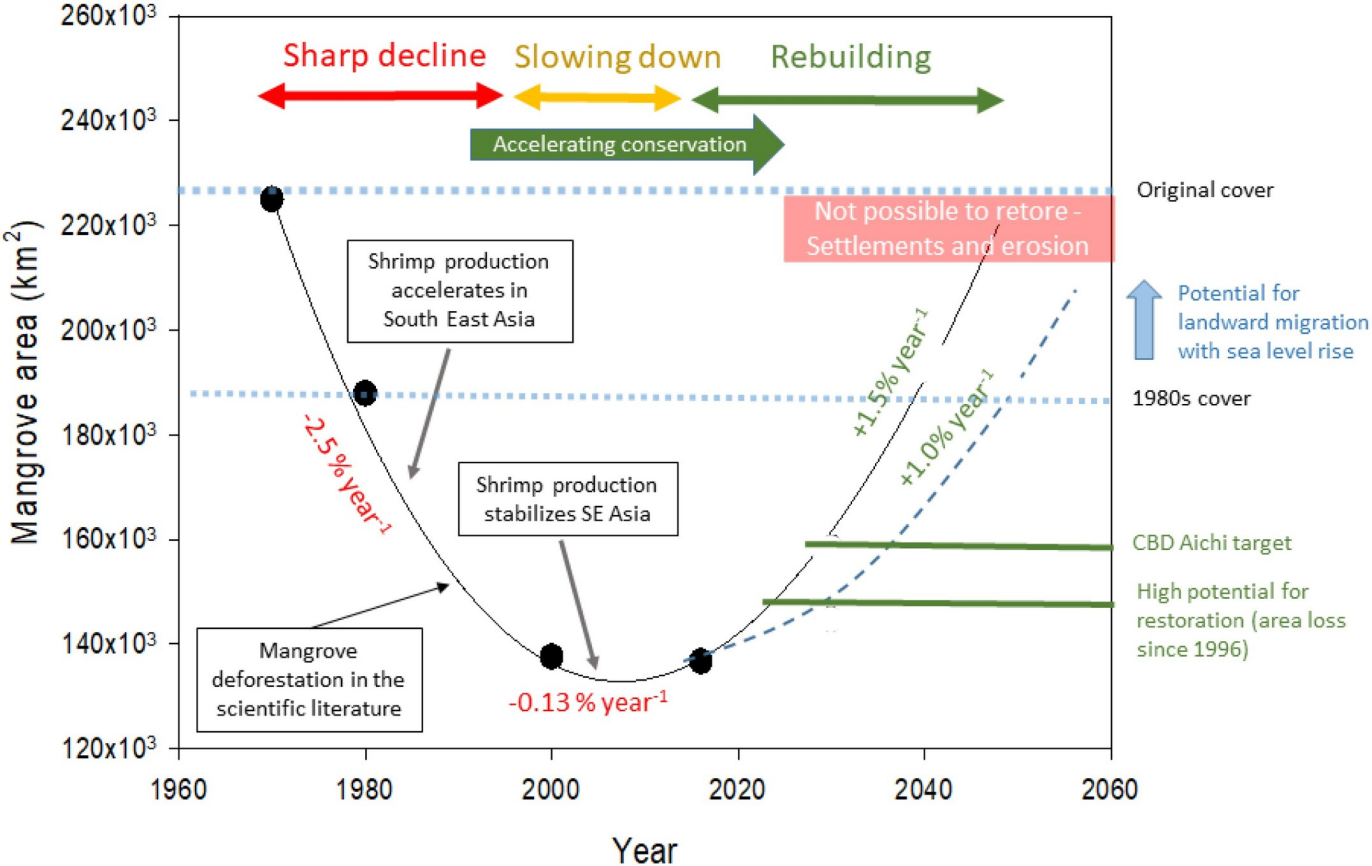
Global trajectories of mangrove cover show a slowdown in the rate of decline. On the right are targets and limits to recovery of mangrove cover. Restoration of approximately 1% year^−1^ will lead to the recovery of high-value sites, achieve Aichi Target 15 by 2040, and return mangrove coverage to 1980s area by 2050 (dashed line). Sea level rise could increase the potential area for restoration with landward migration onto floodplains (blue arrow), while conversion to settlements and erosion will limit restoration potential (red box). Recovery to estimated historical baselines would require approximately 90,000 km^2^ of coastal land.

Mangrove forests have declined globally from an estimated 225,000 km^2^ in the 1970s to about 137,000 km^2^ in 2014 [1]. This decline has led to adverse impacts on the livelihoods of coastal communities; reduced coastal protection leading to loss of property, lives, and infrastructure with extreme events; diminished fisheries; increased social conflict; reduced carbon sequestration capacity while increasing greenhouse gas (GHG) emissions; and decreased nutrient cycling and other important ecosystem services, including those that support resilience of adjacent coral reefs and seagrass meadows [2]. Deforestation and degradation of mangroves also threaten many species that depend on mangrove habitats and their productivity [3]. However, the good news is that between 2010 and 2016, global loss rates slowed down to approximately 0.13% per year [4] (Fig 1). Although deforestation and degradation continue in many countries and regions, mangrove forests are expanding in some areas, including French Guiana, Honduras, the Niger Delta [5], the Red Sea [6], and the Arabian Gulf [7], providing hope for the future. While conservation of the remaining global mangrove cover is immensely important [8], there is also an emerging focus on rehabilitation and restoration (see Box 1 for definition of terms) of mangroves to meet a range of national and international targets [9], particularly during the United Nations Decade of Ecosystem Restoration (2021 to 2030) [10].

**Box 1. Definitions of key terms**

**Rehabilitation**

The process of reinstating a level of ecosystem functionality on degraded sites where ecological restoration is not the aspiration, as a means of enabling ongoing provision of ecosystem goods and services.

**Restoration**

The process of assisting the recovery of an ecosystem that has been degraded, damaged, or destroyed.

**Afforestation**

Artificial establishment of forest on lands, which previously did not carry forest with a living canopy.

**Replanting or Reforestation**

Reestablishment of forest through planting and/or deliberate seeding on land classified as forest.

**National Determined Contributions (NDC) to the Paris Agreement**

NDCs are at the heart of the Paris Agreement and the achievement of these long-term goals. NDCs embody efforts by each country to reduce national emissions and adapt to the impacts of climate change.

### Restoration targets

Targets for restoration influence government and institutional policies and activities [11]. There are numerous international targets for restoration relevant to mangroves and other coastal wetlands. These include the following: the Convention on Biodiversity Aichi Target 15, which calls for “restoration of at least 15 percent of degraded ecosystems by 2020”[12]; the Bonn Challenge with its target of restoring 60 million hectares (600,000 km^2^) of degraded and deforested land to productive, functional, and biodiversity-friendly landscapes [13]; the Trillion Trees by 2050 initiative; the Global Mangrove Alliance target, which aims to conserve and restore mangroves; and the World Wildlife Fund for Nature’s target of restoring 10,000 km^2^ of mangroves. Additionally, Indonesia, the nation with the largest mangrove cover, announced a target of increasing mangrove area by 6,000 km^2^ by 2024 [14]. Countries have also included restoration of coastal wetlands, including mangroves, within their commitment to climate change mitigation and adaptation within their National Determined Contributions (NDC) to the Paris Agreement [15].

Despite the ambitious nature of the restoration targets, mangrove restoration has often been fraught with failure [16]. These failures are mainly due to short-term, large-scale mangrove planting and afforestation programs, which have been conducted in inappropriate or unsuitable environmental locations with inadequate community support [17]. Additionally, some earlier large-scale successful restoration efforts, such as in the Mekong Delta, have been subsequently partially undermined by changing economic policies that favor production of commodities, like shrimp aquaculture production.

Such experiences have led to a range of best practice mangrove restoration guidelines [18]. While mangrove restoration is technically feasible in appropriate locations [19], projects often fail because they have been implemented in unsuitable sites, have foregone best practices to reduce costs through rapid implementation to meet funding deadlines, and may target large numbers of mangrove seedlings planted and not successful establishment. For example, planting mangroves on unsuitable areas, such as low intertidal mudflats or seagrass meadows, is easier and less costly than restoring disused aquaculture ponds [16]. Successful restoration also requires commitment from the communities that manage, use, and interact with mangroves, which can require significant investment in community capacity building and the development of alternative livelihood opportunities [20] that is vital to ensuring the long-term maintenance of restored mangroves. Consequently, one of the main challenges to achieving ambitious global restoration targets will be to depart from the past practice of large-scale planting programs in inappropriate locations, as well as a focus on reducing the underlying pressures that lead to mangrove deforestation.

The complexity of factors influencing restoration success and failure necessitates a broad suite of actions in response [20,21]. To unpack this complexity, we describe different types of restoration opportunities and some of the limitations associated with those opportunities, as well as the strategies developed to identify the most feasible courses of action for restoration of mangrove cover; outline some of the challenges and solutions to scaling up restoration, including options for funding restoration; and describe how progress toward restoration targets may be assessed in ways that simultaneously account for the benefits gained from mangrove restoration and meets multiple policy goals. Adequate monitoring, reporting, and verification is important for characterizing the return on investment in restoration and enhancing understanding of how best to achieve restoration targets.

### Identifying restoration opportunities

The success of mangrove restoration projects is dependent on both socioeconomic and environmental factors that characterize different types of land. The types of land that may be available for mangrove restoration include the following:

1) Former mangrove land that is degraded and fragmented, but where the hydrological processes in the landscape have not been severely changed and tidal flows, which bring sediment, nutrients, and mangrove propagules (seeds and seedlings), are unaltered.

Degraded and fragmented mangrove landscapes (which were formally mangroves) are likely the easiest to restore, providing the underlying causes of degradation are addressed [22]. Currently, there are no global maps of variation in mangrove condition that would allow estimates of the area of mangrove that is degraded and could be restored to further enhance ecosystem functions and services, although fragmentation of mangroves is widespread [23]. New high-definition remote sensing capabilities may allow these areas to be identified in the future facilitating targeted restoration activities.

2) Land that was formally a mangrove but has been converted to “nonproductive” land uses, for example, for housing, infrastructure, and other urban uses.

Typically, for this kind of conversion, tidal flows are excluded, and land is filled before buildings are constructed. This type of land is probably unrestorable [22]. In Southeast Asia, about 2% to 4% of mangrove area has been converted to urban settlements since 2000 [24], and, thus, of the approximately 90,000 km^2^ of global mangrove cover lost over the last century, potentially 4,000 km^2^ is likely unsuitable for restoration (Fig 1).

3) Land that was formally a mangrove (or other wetland type) but has been cleared and converted for production of commodities (e.g., rice, sugarcane, aquaculture, grazing, oil palm, or salt) [24]. Conversion usually involves excluding or altering tidal flows through some kind of modification of the landscape (e.g., drains, channels, levees) or infrastructure (e.g., tidal gates).

There is approximately 9,400 km^2^ of mangrove land that has been converted to commodities since 1996, which may be potentially restorable [22] (Fig 1). If achieved, this would reach approximately half of the Aichi Target 15 of restoring 15% of mangrove area and would be on a trajectory of approximately 1% increase in global mangrove area per year. Recent modelling analyses indicate that this level of restoration is likely achievable by 2050 [11].

However, the amount of mangrove land converted before 1996 that could be restored has not been established [25]. To assess the restoration potential of this earlier cleared land requires knowledge of the current land-use (and income from that land-use), the goals of the communities that own and manage the land, options for acceptable alternative livelihoods, as well as potential future impacts of climate change on commodity production and the restored ecosystems. Restoration of low productivity aquaculture ponds has been recommended for decades. Shrimp farm productivity has declined in many locations due to disease and poor water quality, often leading to the abandonment of up to 90% of ponds within a landscape [26]. This type of converted mangrove land may represent the largest opportunity for restoration of global mangrove cover.

There are cases when converted mangrove land may not be easily restored. Converted mangrove land on exposed coastlines can be highly vulnerable to erosion [27], which is predicted to increase as sea level rise accelerates. The global extent of eroded of mangrove land is unknown, but 30% of losses of mangroves between 2000 and 2016 [4] was attributed to erosion and may therefore be impossible to restore, without engineering solutions. Methods for restoring mangroves on eroded coastlines are emerging [28], but high costs of building and maintaining structures in the intertidal zone may limit their use to sites with particularly high value.

4) Land adjacent to the intertidal zone that is inundated by extreme tides and will become suitable for mangrove growth in the future with sea level rise.

This type of land may currently be used for agriculture or aquaculture or other land-uses and may include other coastal ecosystems (e.g., freshwater ecosystems, or sabkha, saltmarsh with sparse halophytes and microbial mats). A model of future global coastal inundation indicated that sea level rise could increase the land area available for mangroves on floodplains by 20,000 km^2^ by 2100, if human development on floodplains (e.g., from infrastructure) does not prevent mangroves from migrating landward to occupy low elevation land [29]. Rice agriculture on coastal floodplains, which occurs commonly in Southeast Asia and in other tropical and subtropical deltas, is increasingly influenced by salinization due to failing infrastructure, sea level rise, reduced river flows, and drought [30]. While salinized rice land can transition to aquaculture uses [31], mangrove restoration or afforestation could be attractive in locations where agriculture has been adversely affected by saltwater intrusion. In the Arabian Peninsula, extensive coastal sabkha are exposed to increasing inundation with sea level rise and may transition to mangrove forests in the future [32]. Opportunities to accelerate this transition are being explored so the benefits, particularly carbon removal, are collected now and not in a distant future. The suitability of low-lying land for mangrove expansion will depend on the benefits provided by mangroves to communities and landholders, balanced against potential changes in biodiversity, and the ease and cost with which the landscape can be made suitable for mangrove growth (e.g., breaching of levees or modification of drainage works to allow tidal flows).

There is a wide range of factors that might influence the attractiveness of mangrove restoration over the production of commodities, including the risks to agricultural production (e.g., from drought or soil salinization), the development and availability of new salt-tolerant agricultural and aquaculture species; as well as the strength of commodity markets, government support, landholder preferences, and levels of incentives for restoration, including payments for blue carbon and other ecosystem services. While these are complex interacting factors, there are methods available to help plan restoration. For example, the Restoration Opportunities Assessment Methodology (ROAM) [33] identifies the opportunities for ecosystem restoration while considering environmental factors and social contexts.

### Managing negative effects of climate change on restoration

While sea level rise may provide some opportunities for increasing mangrove cover, potential losses of increasingly inundated seaward fringing mangroves due to sea level rise and erosion is predicted to be approximately 5,000 km^2^ by 2100 [29] (Fig 1). Other potentially negative impacts of climate change on the restoration of global mangrove cover include increasing fluctuations in sea level, drought, changing ocean–climate cycles that accelerate erosion, and changes in frequency of intense storms and other extreme events [34]. The strength of these additional factors on mangrove cover and condition, and interactions with anthropogenic stressors (e.g., coastal development and eutrophication) are likely to be spatially variable [35], with the consequence that climate change effects on restoration projects are also spatially variable and require local assessments and planning.

Several strategies have been identified to reduce climate risks to restoration projects. These include selection of sites in locations with lower exposure to extreme events [22]. For example, a focus on estuarine or lagoon settings compared to open coasts, which are exposed to wind and waves during intense storms. Maintaining sediment and freshwater supply and other conditions that support mangrove growth and long-term survival (e.g., reduced pollutants) [36], including connections to existing mangroves that provide sources of propagules and habitat for fauna, are also important for enhancing resilience to climate risks [23]. Choosing suitable species that are resilient to storm damage or other environmental factors can also enhance restoration success with extreme events [37]. For example, *Avicennia* sp. have a high capacity to recover after intense storms compared to *Rhizophora* sp., which are commonly planted because they are easy to collect and plant but are less tolerant of storm damage.

Planning activities to enhance recovery after extreme climate events is also important for reducing climate risks. Where restoration projects are supported by payments for ecosystem services, projects could be designed using adaptive management to accommodate climate risks [38,39]. For example, the Sundarbans, the largest mangrove in the world, has recently experienced major losses due to cyclones. Restoration efforts could be deployed following cyclone impacts and other climatic and human damage to vegetation to avoid CO_2_ emissions from denuded sediments and restore the carbon sink [40]. Accelerating early phases of mangrove regeneration could be effective. For example, the use of “nurse” plants and other novel techniques to facilitate mangrove seedling establishment [38,39], including emerging techniques such as plant and microbe manipulations to restore polluted soils and improve mangrove growth and health [41].

### Scaling up restoration

#### One size does not fit all—Finding the “sweet spot” in the scale of mangrove restoration projects

Since the 1970s, some large mangrove reforestation and afforestation projects have demonstrated that mangrove restoration at the scale of thousands of hectares is feasible, particularly with strong government support and in landscapes with unaltered tidal flows. One advantage of such projects is that they can deliver important landscape-scale benefits, such as flood mitigation and carbon sequestration (Table 1). For example, the Mekong Delta is the site of the largest ecosystem restoration ever undertaken. About 55% of the Mekong Delta mangrove forest was destroyed in the USA-Vietnam war between 1964 and 1970 through use of the herbicide Agent Orange [42]. The Vietnamese subsequently restored 1,500 km^2^ of mangrove in the Delta between 1978 and 1998 [42], planting on average about 6,000 ha per year. The planted forest has a carbon stock estimated at 889 ± 111 Mg C ha^−1^, comparable to that reached by natural forests [43]. The CO_2_ sequestered by the restored mangroves of the Mekong Delta (approximately 24 million tonnes CO_2_ equivalents per year) represents up to 10% of the annual emissions reported by Vietnam (251 million tonne CO_2_ equivalents in 2012). The restored mangrove also forms the basis of tourism and fisheries industries [44]. Additional examples of large-scale planting programs that have increased mangrove cover include the mangrove restoration and afforestation in the Arabian Gulf and those in Bangladesh [45]. In the UAE, ambitious mangrove planting programs, started in the 1970s, doubled the mangrove area in the UAE [7], with these efforts continuing under the Mangrove National Park protected by the Environment Agency of Abu Dhabi.

**Table 1:** Summary of the benefits and problems associated with larger and smaller mangrove restoration projects.

Project size	Potential benefits	Potential problems
Larger projects	• Potential for rehabilitation and restoration of ecosystem services over large scales • Economies of scale • Attractive to investors • Can support high levels of biodiversity • Landscape scale ecosystem service provision	• Inappropriate biophysical conditions • Limited engagement with large number of stakeholders • Complex governance • Failure to address underlying causes of degradation • Monospecific plantings • Large but short-term investment
Smaller projects	• Empowerment of landholders/communities • Simple governance • Appropriate sites selected by landholders • Addresses underlying causes of degradation and allows experimentation • Long-term commitment to land management	• Small patches may not deliver ecosystem services • Higher costs of implementation per area of habitat • Unattractive, and often invisible, to investors • Limited biodiversity benefits

Large-scale mangrove restoration and afforestation projects have been particularly prevalent as a response to natural disasters. However, like major infrastructure projects, such large-scale planting projects have been vulnerable to a range of risks [46] (Table 1). For example, the Indian Ocean tsunami of 2004 led to large-scale mangrove restoration and afforestation projects by both governments and international nongovernment organizations (NGOs), initiated to increase coastal protection, many of which failed because of the overemphasis on area-based planting targets (often associated with afforestation) over best-practice restoration [16]. In some cases, these projects led to cycles of dependency of local communities on international aid funding for replanting mangroves [47]. In Myanmar, the government has led the replanting of about 1,000 ha of mangroves per year after Cyclone Nargis caused high levels of fatalities in the Ayeyarwady Delta, but overexploitation and loss of the mangrove have continued because the underlying causes of deforestation have not been alleviated [48].

The importance of mangroves for delivering ecosystem services and the learnings from earlier restoration failures have stimulated the development of other types of large projects that comprise a range of activities including conservation, rehabilitation, and development of alternative livelihoods. For example, the Cispata blue carbon project in Colombia includes 11,000 ha of protection and rehabilitation of mangroves, and in Indonesia, the Mangroves for Coastal Resilience Project, supported by the World Bank, plans to restore 75,000 ha of mangroves along with implementation of a range of other measures that include conservation, livelihood improvements, capacity building, and reforms in governance and policy.

Large-scale projects are not the only pathway to successful mangrove restoration. Smaller-scale mangrove restoration projects (<1,000 ha) have higher rates of plant survival than larger projects [49] (Table 1). This occurs because smaller projects are often done by motivated landholders and community groups (e.g., the 117-ha Mikoko Pamoja project in Kenya [50], or 392 ha of shrimp pond restoration on Tanakeke Island in Indonesia [51]) and reflect concentrated management effort, even with limited resources. Smaller projects may work particularly well in contexts where sustainable extraction of resources from the mangroves is important for local communities [52].

The disadvantages of restoring small and potentially isolated patches of mangroves are that they are fragmented and may offer little benefits for biodiversity, particularly for large mangrove-associated species that require large home ranges, or other ecosystem benefits [23] (Table 1). Although, they may also form connected patches over which larger populations are connected. Many ecosystem services increase nonlinearly across landscapes and are dependent on achieving a critical habitat size, for example, support for coastal and marine fisheries and coastal protection [21]. Smaller projects may be more expensive per hectare to implement than larger projects, although a review of restoration costs did not detect this [49]. Finally, small community-managed projects can make their own decisions about where to spend any income from projects, which may not necessarily align with the targets of funders.

Reconciling the advantages and disadvantages of large versus small restoration projects could be achieved through nesting smaller, community-led projects into larger projects. Government-supported schemes, such as community forestry nested within coastal adaptation planning, could help communities implement smaller restoration projects while also delivering landscape-scale benefits [50]. In small island states, which are particularly vulnerable to climate change, small restoration projects may provide the most appropriate scale and deliver the most benefits in providing a range of ecosystem services and supporting community livelihoods [51]. Aggregating multiple small projects through the establishment of collaborative networks could have additional benefits of increasing attractiveness for funders (see below) and facilitating learning and exchange of ideas among communities, while also reducing the costs of maintaining key infrastructure, such as mangrove nurseries and costs of verification for carbon-based projects, through economies of scale. This kind of approach could improve mangrove restoration over a range of local settings, thereby also leading to capacity building and increasing the adaptive capacity to climate change of communities.

#### Funding scaled-up mangrove restoration

Payments for ecosystem services have been widely proposed to support mangrove restoration. Payments for abatement of CO_2_ emissions or blue carbon have become the impetus for funding mangrove restoration projects, and some large-scale (>5,000 ha) blue carbon projects have been developed for the voluntary carbon market, for example, in Indonesia, Pakistan, Senegal, and Colombia. Returns on investment are likely to be high and should be evaluated further [52]. While the long-term success of these projects is yet to be proven, some concerns have been raised on equitable sharing of benefits among external partners and communities as well as among community members [53]. Similar to terrestrial carbon projects, blue carbon payment schemes carry risks for communities. Currently, the establishment and verification costs of blue carbon projects under carbon standards (e.g., carbon projects within VERRA, Gold Standard, or Plan Vivo) are high, necessitating large project sizes to offset costs [54] that may then incur the risks associated with large projects (Table 1). Additionally, independent of project size, restoration projects that plan to sell carbon credits internationally (as carbon offsets) are exposed to uncertainty as nations decide on which country (the nation of the buyer or the seller) can count these toward their NDCs and thereby avoid double counting of carbon sequestered.

Solutions to the high cost of payment for ecosystem services (PES) schemes have included funding by philanthropic donors and other sources [55]) and designing schemes with lower costs or high payments for benefits (e.g. carbon + biodiversity). Mangrove restoration projects that seek payments for carbon sequestration and avoided emissions on the voluntary market require registering of projects and verification of outcomes. Measuring and monitoring carbon sequestration are complex activities and often require communities to collaborate with NGOs, private companies, research institutions, or governments to successfully prepare the documentation and implement projects. Developing low-cost but robust methodologies could help further incentivize restoration [56]. Governments have been encouraged to include mangroves and other coastal wetlands in their national GHG inventories (e.g., Conference of the Parties 25, Madrid 2019), a policy that would favor development of robust national methods for accounting and restoration actions, but most have not done so yet [11]. However, if widely adopted, national policies linked to national GHG reduction targets could boost mangrove restoration for their role in contributing to GHG reductions.

Blue carbon payments on their own may be insufficient to fund restoration of mangroves. Consequently, payments for additional ecosystem services, for example, coastal protection, nutrient cycling, biodiversity conservation and fisheries, and assessment of returns on investment are needed to incentivize scaled-up restoration. The development of multiple-use systems to increase mangrove cover in landscapes while maintaining landholder incomes is an attractive option in some countries (e.g., certified organic shrimp projects). In Bangladesh, such systems generated benefit-to-cost ratios of 1.0–2.7 [57]. However, multiple-use systems often provide less biodiversity and other ecosystem services (coastal protection) because of their fragmented nature, and in the Mekong Delta, payments for organic shrimp have been low [58]. Payments for reducing fertilizer inputs aimed at improving water quality are emerging (e.g., Reef Credits). Similar schemes where landholders could receive payments for nutrient retention and cycling through mangroves restoration could increase the incentives for landholders to restore mangroves and other coastal wetlands.

A growing number of Environment, Social, and Governance (ESG) funds seek opportunities to fund projects that achieve climate change mitigation while contributing to advancing UN Sustainable Development Goals (SDGs). Businesses may also have Corporate Social Responsibility (CSR) targets that may incentivize funding for restoration. However, there is, at present, a mismatch between the availability of projects on the ground, often in small communities in developing nations, and the available funding from impact investment funds, which typically finance projects worth millions of dollars [59]. Better alignment of funding with mangrove restoration projects could be achieved through establishing clearing houses whereby smaller projects could be aggregated into a single portfolio for investors. For example, common asset trusts [60] could maintain an inventory of funding opportunities for mangrove restoration projects. These projects would be endorsed by local stakeholders and adhere to social safeguards, including comanagement arrangements with communities and fair benefit sharing [50]. Such arrangements offer ESG investment funds a range of options with clearly articulated benefits to communities that can support long-term investment plans. The UN maintains an inventory of projects offering UN Certified Emission Reductions (CERs) located in developing countries, where CERs can be purchased to offset unavoidable emissions or as a contribution to global climate action. While this is a promising initiative, no mangrove restoration or conservation projects are currently offered [61], so the gap between financial resources available and the offering of viable projects on the ground continues to grow.

## Benefiting from tracking and reporting on restoration outcomes

Unbiased monitoring of the outcomes of restoration has a multitude of benefits for stakeholders at both the project level and larger regional and national scales. Importantly, monitoring and evaluation during restoration enables adaptive management, including evaluation of the effectiveness of different restoration actions and techniques. The Society for Restoration (SER) has long advocated for clear targets and detailed monitoring and reporting on restoration projects to learn from project failures and successes [62]. SER recommends that reporting should not only include biological and environmental outcomes but also evaluate socioeconomic outcomes for communities. Project-level reporting is increasing with adoption of SER and/or other international standards, and data streams relevant to mangroves at larger scales are emerging along with novel techniques, including monitoring annual change with remote sensing [63] and citizen-science approaches to monitoring. Standard reporting methods for mangrove restoration projects are under development, but reporting on restoration outcomes for mangroves at larger spatial scales (e.g., subnational and national) against targets is not yet developed but needed. Increased scientific capacity to plan, implement, monitor and, report on restoration projects, so that the local communities are fully capable to implement all project phases, should be a high priority.

Restoration projects that aim to enhance carbon storage among their goals involve a commitment to ensure the permanence required to achieve the climate mitigation goals, usually 100 years. Ensuring permanence over many decades needs transgenerational commitments from communities and funding bodies as well as governance to support long-term stability of mangrove restoration projects, for example, to secure land for restoration conditions could be placed on land titles [64]. Sea level rise and other climate change impacts will also affect the long-term stability of restored mangroves (discussed above). Mangrove restoration should plan for changing distributions of mangroves with sea level rise (e.g., landward migration), making necessary arrangements over the land needed for landward migration [55].

## Aligning mangrove restoration in national reporting

Verification of landscape-scale ecosystem restoration enables evaluation of progress against targets. Currently, there is no national-level mangrove-specific reporting (although nations can report on GHG emissions from coastal wetlands to the UN Framework Convention on Climate Change (UNFCCC); see below) (Table 2). Nations report on restoration through the Global Biodiversity Framework Convention on Biological Diversity on Aichi Target 15 (Resilience and Restoration) against indicators including the primary indicators “Proportion of land that is degraded over total land area” and “Bioclimatic Ecosystem Resilience Index (BERI)”. The proportion of land that is degraded could be adapted to report changes in mangrove cover (which could reflect restoration success), but this is not explicitly required. For example, Fiji reported change in mangrove cover against Target 15 and in its National Biodiversity Strategy and Action Plan [65]. However, only terrestrial species are reported for BERI at the global scale. Other proposed reporting indicators include the “Ocean Health Index” [66], which, while having a mangrove component, uses older data sets and is an index that aggregates over multiple ecosystems, and, therefore, is unlikely to be updated sufficiently frequently to serve the purpose of tracking outcomes of mangrove and other coastal wetland restoration. Other secondary indicators (e.g., Wildlife Picture Index, Cumulative Human Impacts on Marine Ecosystems, Human Appropriation of Net Primary Production (HANPP)) also have some potential for reporting on mangrove restoration. Reporting on mangrove restoration can also be made against Strategic Goal E (Enhance implementation through participatory planning, knowledge management, and capacity building, Aichi Targets 17 to 20). New solutions for reporting are on the horizon. The post-2020 Biodiversity Framework (OECD 2019; [67]) recommends that area of mangrove extent could be a potential indicator (Table 2), which could provide a direct measure of restoration outcomes, and is feasible given the high level of data availability [60]. Additionally, the benefits of mangrove restoration could be reported in the Environmental Economic Accounts indicator (Table 2), allowing further assessments of the benefits derived from investments in mangrove restoration.

**Table 2:** National reporting on mangrove restoration or the impacts of mangrove restoration and current challenges in the adequacy of reporting frameworks and potential solutions.

Convention/Treaty	Characteristics	Challenge	Solution
UN Framework Convention on Climate Change (UNFCCC)	Emissions associated with a range of activities in coastal wetlands, including mangroves	Limited countries report on emissions from coastal wetlands. Mangroves may be included in Forests category or within Wetlands.	More assistance for countries to use the IPCC guidance, capacity building for policy and reporting
Global Biodiversity Framework Convention on Biological Diversity (CBD), Aichi Targets 10, 15, 17	Forest cover, Bioclimatic Ecosystem Resilience Index (BERI) Various composite indices (e.g., Ocean Health Index)	Indices are not mangrove focused but focus on terrestrial ecosystems, species based or composites of a range of biological and physical properties and threats	Report on change in mangrove extent (adopt proposed Post-2020, A.0.1, and Species Habitat Index, A.0.2) and condition; Report benefits using National environmental economic accounts of ecosystem services (adopt proposed in Post-2020 B.0.1); capacity building to implement globally accepted tools
Sustainable Development Goals, Goal 14 Life below water, 14.2 Protect and Restore Ecosystems	Ocean acidification, Area protected, Fisheries	Tend to report on fisheries and development rather than extent and condition of mangroves and other coastal wetlands.	Report on change in mangrove and other coastal wetland extent and condition; capacity building and develop globally accepted tools
Ramsar convention Target 8 National wetland inventories have been either initiated, completed, or updated and disseminated and used for promoting the conservation and effective management of all wetlands (8.6 extent of wetlands)	Report on extent of mangroves in designated sites	Many countries do not have national wetland inventories that are updated over time	Develop national wetland inventories with monitoring, capacity building.
UNESCO World Heritage	Criteria of Outstanding Universal Value (cultural, ecological, geological)	May or may not include data on extent of mangroves or other coastal wetlands	Develop national wetland inventories with monitoring; capacity building.
Sendai Framework for Disaster Risk Reduction	Reporting on exposure to risks and activities to reduce risks (e.g., risk reduction plans)	Focused on human and economic losses. Targets E and F focus on plans to reduce risks and international capacity building.	Include indicators that reflect investments in Nature-based Solutions

National reports to the UNFCCC Paris Agreement include land-use change and can include reporting for changes in carbon stocks associated with changing cover of mangroves, tidal marshes, and seagrass. However, only a limited number of countries have used this option, although many more nations include mangrove management for climate change adaptation within their NDCs [11]. Increasing uptake of reporting of emissions from coastal wetlands using IPCC guidance could be facilitated by enhanced national commitments to GHG reporting, and capacity building efforts, including, for example, “how-to manuals” for GHG inventory compilers [68].

National reports to the Ramsar Convention on Wetlands of International Importance and UNESCO World Heritage sites, although focused on a limited number of designated sites, rather than at a national level, could also be used to report on mangrove restoration. National reports on mangroves could also be made within the SDGs framework on SDG 14 (life underwater) in the Voluntary National Reviews (VNR). Target 14.2 refers specifically to restoration of coastal ecosystems: “Sustainably Manage and Protect Coastal Ecosystems, Including Their Restoration”. However, the VNRs tend to comprise high-level aspirations focused on development issues (e.g., industry and communities). For example, in the 2017 VNRs, only 11 of 118 countries with mangroves mentioned SDG 14 in their executive summaries. Of these, 10 focused predominantly on the role in fisheries, reflecting the dominant focus on development within the SDGs. Similarly, the Sendai Framework for Disaster Risk reduction is focused on economic losses, but could increase focus on Nature-based Solutions, including restoration of mangroves and other coastal ecosystems.

Streamlining the reporting on restoration across these disparate UN frameworks would greatly help to characterize the benefits of restoration. With greater alignment, countries with strong and appropriate mangrove restoration programs could report increases in mangrove habitat to Achi Target 15, reduced carbon emissions within NDCs, biodiversity and social benefits within SDGs, and coastal protection through the Sendai Framework. All of these reporting pathways are under UN conventions, which can be aligned and resourced to achieve high-level coordination, and thereby avoid redundant, but partial, reporting efforts.

## Conclusions

Mangrove restoration (and restoration of all coastal wetlands) is highly desirable for the ecosystem services they deliver. Global mangrove restoration targets reflect the ambition for restoring the global extent of mangroves, and the technical knowledge needed for successful restoration of mangroves is strong and increasing. However, past performance of restoration has revealed failures that may be repeated unless a wide range of interventions and new processes are adopted. These include synthesizing both environmental (including climate change) and socioeconomic conditions influencing opportunities for mangrove restoration. The use of frameworks that consider both components can help identify land that is suitable and feasible for restoration. Establishing restoration targets that recognize that some lands that were previously mangrove or that are exposed and affected by extreme climate events may be impossible to restore. Targets should be feasible and reflect permanent landscape changes.

Developing mechanisms to support small, community-led restoration projects are important for long-term sustainability of restoration. Community-led projects are highly desirable, but to achieve landscape-scale and widespread community benefits and to attract funding, mechanisms to aggregate small projects are needed. The design of simple, low-cost, and robust methodologies for characterizing ecosystem services from restoration could decrease costs and increase uptake of payments for ecosystem schemes, which could further help to implement mangrove restoration. Long-term, adequate, and dependable financial support and governance arrangements are needed to ensure long-term successful mangrove restoration. Adoption of appropriate methods for reporting on mangrove restoration to support adaptive management and enable countries to report on obligations to multiple international agreements and treaties that reflect the multifaceted value of mangrove restoration will increase interest in and incentives for mangrove restoration.

The past destruction of mangroves and the growing impacts of climate change make restoring mangroves and other coastal wetlands an urgent global need. Given the scope of the interventions required to support ambitious mangrove restoration (i.e., the 1.5% increase in cover per year for 30 years), the development and adequate resourcing of a global mangrove restoration program, comparable to than in place for coral reefs, and building on recent initiatives like the Blue Carbon Accelerator Fund, would propel the scaling-up of appropriate restoration efforts as well as the development of unified frameworks, such as cost-effective project design and verification systems. Such a program would benefit many regions of the world with limited capacity and provide a platform for developing a portfolio of regionally appropriate solutions for mangrove restoration.

## Abbreviations

BERI: Bioclimatic Ecosystem Resilience Index
CER: Certified Emission Reduction
CSR: Corporate Social Responsibility
ESG: Environment, Social, and Governance
GHG: greenhouse gas
HANPP: Human Appropriation of Net Primary Production
NGO: nongovernment organization
NDC: National Determined Contributions
PES: payment for ecosystem services
ROAM: Restoration Opportunities Assessment Methodology
SDG: Sustainable Development Goal
SER: Society for Restoration
UNFCCC: UN Framework Convention on Climate Change
VNR: Voluntary National Review
